# Supplementary material for: Single-Walled Carbon Nanotubes Attenuate Cytotoxic and Oxidative Stress Response of Pb in Human Lung Epithelial (A549) Cells
Source: Int J Environ Res Public Health. 2020 Nov 6;17(21):8221. doi: 10.3390/ijerph17218221 (PMC7664418; doi:10.3390/ijerph17218221)
Supplement: Supplementary file 1 [file ijerph-17-08221-s001.pdf]

<u>Supplementary Information</u>

Single-Walled Carbon Nanotubes Attenuate Cytotoxic and Oxidative Stress Response of Pb in Human Lung Epithelial (A549) Cells

Maqusood Ahamed*, Mohd Javed Akhtar, M.A. Majeed Khan

King Abdullah Institute for Nanotechnology, King Saud University, Riyadh-11451, Saudi Arabia

***** Correspondence:

Maqusood Ahamed, Ph.D.

Associate Professor

King Abdullah Institute for Nanotechnology

King Saud University

Tel.: +966114698781

Email: mahamed@ksu.edu.sa

S1. Materials and methods

S1.1. Synthesis of SWCNTs

Single-walled carbon nanotubes (SWCNTs) were grown on a Si substrate (n-type <100>) through plasma-enhanced vapor deposition (PECVD) technique [1]. Firstly, Si substrate was washed in acetone with ultrasonic bath and dried at room temperature. Then, Si substrate was placed in a radio frequency (RF) sputtering chamber for the deposition of Fe catalyst with the power of 100 W. Further, Fe coated Si substrate was placed on the substrate holder of the PECVD system. The chamber was then evacuated to 10-3 torr. Initially, quartz tube was purged several times with nitrogen gas and then Fe coated catalyst substrate was pre-treated in hydrogen (H_2) atmosphere at a constant temperature of 400 ^{0}C. Subsequently, acetylene as a gas with H_2 gas carrier was introduced into the system for 20 minutes at the rate of 25 sccm. During the growth process, temperature and pressure inside the chamber were maintained at 600 ^{0}C and 200 torr, respectively. At the end, PECVD system was switched off and the product was cooled down to the room temperature.

S1.2. MTT assay

Cell viability was measured by MTT assay [2] with some specific modifications [3]. In brief, 20,000 cells/well seeded in a 96-well plate and allowed for 24 h to attach on the surface of plate. Then, cells were treated for 24 h to different concentrations of SWCNTs (0-200 µg/ml) and Pb (0-100 µg/ml). For combined cytotoxicity study, cells were exposed for 24 to either SWCNTs (10 µg/ml) or Pb (50 µg/ml) or combination of both (10 µg/ml SWCNTs and 50 µg/ml Pb). At the end of exposure time, culture medium was removed from each well to avoid interference of SWCNTs and replaced with new medium containing MTT solution in an amount equal to 10% of culture volume. The 96-well plate is now incubated for 3 h at 37 °C until a purple colour formazan product was developed. The resulting formazan product was dissolved in acidified isopropanol. Further, 96-well plate was centrifuged at 2500×g for 5 min to settle down the SWCNTs, if present in the solution. Then, a 100 µl supernatant was transferred to other fresh wells of 96-well plate and absorbance was measured at 570 using a microplate reader (Synergy-HT, BioTek, Vinnoski, VT, USA).

S1.3. Cell cycle analysis

Cell were exposed for 24 h either SWCNTs (10 µg/ml) or Pb (50 µg/ml) or mixture of both (SWCNTs+Pb). After the completion of exposure time, control and treated cells were harvested and centrifuged at 2500×g for 5 min to get cell pellets. Further, cell pellets were re-suspended in 500 µl of phosphate buffer saline (PBS) and fixed with equal volume of chilled 70% ice-cold ethanol, and incubated at 4 °C for 1 h. After two successive washes with PBS cell pellets were re-suspended in PBS and stained with 50 µg propidium iodide (PI)/ml containing 0.1% Triton X-100 and 0.5 mg/ml RNAase A for 1 h at 30 °C in the dark. Fluorescence of the PI was measured by flow cytometry (Beckman Coulter, Coulter Epics XL/Xl- MCL, Miami, USA) through a FL4 filter (585 nm) and 10,000 events were acquired. The data were analyzed by Coulter Epics XL/XL-MCL, System II Software, Version 3.0. Cell debris was characterized by a low FSC/SSC was excluded from the analysis [4].

S1.4. Assay of caspase-3 and caspase-9 enzymes activity

Activity of caspase-3 and -9 enzymes was assayed using commercial kits (BioVision, Milpitas, CA, USA). This assay is based on the principle that activated caspases in apoptotic cells cleave the synthetic substrates to release free chromophore p-nitroanilide (pNA), which was recorded at 405 nm. The pNA produced after specific action of caspase-3 and caspase-9 on tertrapeptide substrates were DEVD-pNA and LEHD-pNA, respectively. In brief, reaction

mixture consisted of 50 µl of control and treated cell extract protein (50 µg), 50 µl of 2X reaction buffer (containing 10 mM dithiothreitol) and 5 µl of 4 mM DEVD-pNA (for caspase-3) or LEHD-pNA (for caspase-9) substrate in a total volume of 105 µl. The reaction mixture was incubated at 37 °C for1 h and absorbance of the product was measured using a microplate reader (Synergy-HT, BioTek) at 405 nm according to manufacturer's instruction.

S1.5. Mitochondrial membrane potential assay

Mitochondrial membrane potential (MMP) was quantified using rhodamine-123 probe (Rh-123, Sigma-Aldrich) [5]. Cell were exposed either SWCNT (10 µg/ml) or Pb (50 µg/ml) or mixture of both (SWCNTs+Pb) for 24 h. Briefly, 20,000 cells/well seeded in a 96-well plate and allowed for 24 h to attach on the surface. After the completion of exposure time, cells were harvested and washed twice with PBS. Cells were further exposed with 10 µg/ml of Rh-123 probe for 1 h at 37 °C in the dark. Again, cells were washed with PBS and fluorescent intensity of Rh-123 was measured at a microplate reader (Synergy-HT, BioTek).

S1.6. Reactive oxygen species generation assay

Intracellular ROS generation was measured using 2,7-dichlorofluorescin diacetate (DCFH-DA) as reported earlier [5]. ROS level was estimated through two procedures; quantitative analysis and microscopic fluorescence imaging. For quantitative assay, cells (20000cells/well) were seeded in 96-well black-bottomed culture plates and allowed to adhere for 24 h in a CO_2 incubator at 37 °C. Further, cells were exposed to either SWCNT (10 µg/ml) or Pb (50 µg/ml) or mixture of both (SWCNTs+Pb) for 24 h. After the completion of exposure time, cells were washed twice with HBSS before being incubated in 1 ml of working solution of DCFH-DA at 37 °C for 30 min. Then, cells were lysed in alkaline solution and centrifuged at 2300×g for 15 min to settle down the cell debris. A 200 µl supernatant was transferred to a new 96-well plate, and fluorescence was measured at 485 nm excitation and 520 nm emission using a microplate reader (Synergy-HT, BioTek). The values were expressed as a percent of fluorescence intensity relative to the control cells. A parallel set of cells in a transparent 96-well plate was analysed for intracellular fluorescence using a DMi8 fluorescent microscope (Leica Microsystems, Leica Microsystems, GmbH, Germany), with images taken at 20X magnification.

S1.7. Preparation of crude cell extract

For the assay of Malondialdehyde (MDA) level, glutathione (GSH) level, and activity of several antioxidant enzymes such as glutathione peroxidase (GPx), superoxide dismutase (SOD), and catalase (CAT) cell extract was prepared. In brief, cells were cultured in 75-cm^2 culture flask and exposed either SWCNT (10 µg/ml) or Pb (50 µg/ml) or mixture of both (SWCNTs+Pb) for 24 h. At the end of exposure time, cells were harvested in ice cold PBS by scraping and washed with PBS at 4 °C. Cell pellets were further lysed in cell lysis buffer [1X 20 mM Tris-HCl (pH 7.5), 150 mM NaCl, 1 mM Na_2EDTA, 1% Triton, 2.5 mM sodium pyrophosphate]. Following centrifugation (15000 g for 10 min at 4 °C) the supernatant (cell extract) was maintained on ice to perform the required experiments.

S1.8. Malondialdehyde estimation

Malondialdehyde (MDA), an end product of lipid peroxidation was estimated using procedures of Ohkawa et al [6]. In brief, a mixture of 0.1 ml cell extract and 1.9 ml of 0.1 M sodium phosphate buffer (pH 7.4) was incubated at 37 °C for 1 h. Then, mixture was precipitated with 5% trichloroacetic acid (TCA) and centrifuged (2500×g) for 15 min to collect supernatant. Furthermore, 1.0 ml of 1% thiobarbituric acid (TBA) was added to the supernatant and placed in the boiling water for 15 min. After cooling to room temperature absorbance of the mixture was taken at 532 nm and was converted to MDA and expressed in nmole MDA/mg protein using molar extinction coefficient of 1.56×10^5 M^-1 cm^-1.

S1.9. Glutathione assay

The glutathione (GSH) level was quantified using Ellman's method [7]. In brief, a mixture of 0.1 ml of cell extract and 0.9 ml of 5% TCA was centrifuged (2500×g) for 15 min at 4 °C. Then, 0.5 ml of the supernatant was added into 1.5 ml of 0.01% 5,5'-dithiobis-(2-nitrobenzoic acid (DTNB) and the reaction was monitored at 412 nm. The amount of GSH was expressed in terms of nanomole/mg protein.

S1.10. Glutathione peroxidase enzyme assay

Activity of glutathione peroxidase (GPx) enzyme was measured using protocol of Rotruck and co-workers [8]. In brief, a reaction mixture contained 20 µl of 0.1 M GSH, 10 µl of cell extract, 100 µl of 2 mM NADPH, 100 µl of 10 U/ml glutathione reductase, 800 µl of 0.2 M Tris-HCl, and 10 µl of 5 mM t-butylhydroperoxide. The oxidation rate of NADPH was monitored at 320 nm.

S1.11. Catalase enzyme assay

Enzymatic activity of catalase was assayed according to the protocol of Sinha et al [9]. In this method, dichromate in acetic acid is reduced to chromic acetate in the presence of H_2O_2. Briefly, reaction mixture (150 µl) contained 100 µl of 0.01M phosphate buffer, 10 µl cell extract, and 40 µl of 2M H_2O_2 was prepared. The reaction was stopped by mixing of 200 µl of dichromoacetic acid reagent (5% of potassium dichromate and glacial acetic acid in 1:3 ratio) and absorbance was recorded at 530 nm.

S1.11. Interaction of Pb and SWCNTs in culture medium

Adsorption of Pb on the surface of SWCNTs in complete cell culture medium (DMEM+10%FBS) was determined by inductively coupled plasma mass spectrometry (ICP-MS) [10]. Briefly, samples were categorized into three groups (n=3); Pb group (50 µg/ml Pb in culture medium), co-exposure group (10 µg/ml SWCNTs and 50 µg/ml Pb in culture medium) and control group (only culture media). All three samples were incubated for 0 and 24 h with gentle shaking. Then, supernatant was collected after high speed centrifugation. Supernatants were further digested with nitric acid. The digested solution was further dissolved in 4% nitric acid and Pb content was measured by ICP-MS. The adsorbed amount of Pb on the surface of SWCNTs was equal to decreased level of Pb in supernatant over 24 h.

S1.12. Effect SWCNTs on cellular uptake of Pb

Effect of SWCNTs on cellular uptake of Pb was also measured by ICP-MS [10]. Briefly, 20000 cells/well cultured in 96-well plate and allowed 24 h to attach on the surface. Then, cells were exposed for 24 to 50 µg/ml of Pb with or without SWCNTs (10 µg/ml). After the completion of exposure time, cells were washed several times with PBS and harvested. Then, harvested cells were digested in nitric acid. The digested solution was further dissolved in 4% nitric acid and Pb content was measured by ICP-MS. The intracellular level of Pb was presented in the unit of picogram (pg) per cell.

S2. Results

Figure S1 showed the attenuating effects of different concentrations of SWCNTs (1-200 µg/ml) against Pb-induced cytotoxicity (50 µg/ml) in A549 cells. We can see that SWCNTs at a concentration of 10 µg/ml achieved maximum attenuation effect against 50 µg/ml Pb induced cytotoxicity in A549 cells. Above the concentration of 10 µg/ml SWCNTs attenuating effects against Pb induced toxicity was not much different. This indicated that 10 µg/ml SWCNTs were enough to adsorbed most of Pb (50 µg/ml) present in the culture media.

Previous *in vitro* and *in vivo* studies also demonstrated that pure SWCNTs at the concentration of 10 µg/ml did not induce toxicity [11]. Hence, for further co-exposure experiments we have chosen this concentration (10 µg/ml) of SWCNTs.

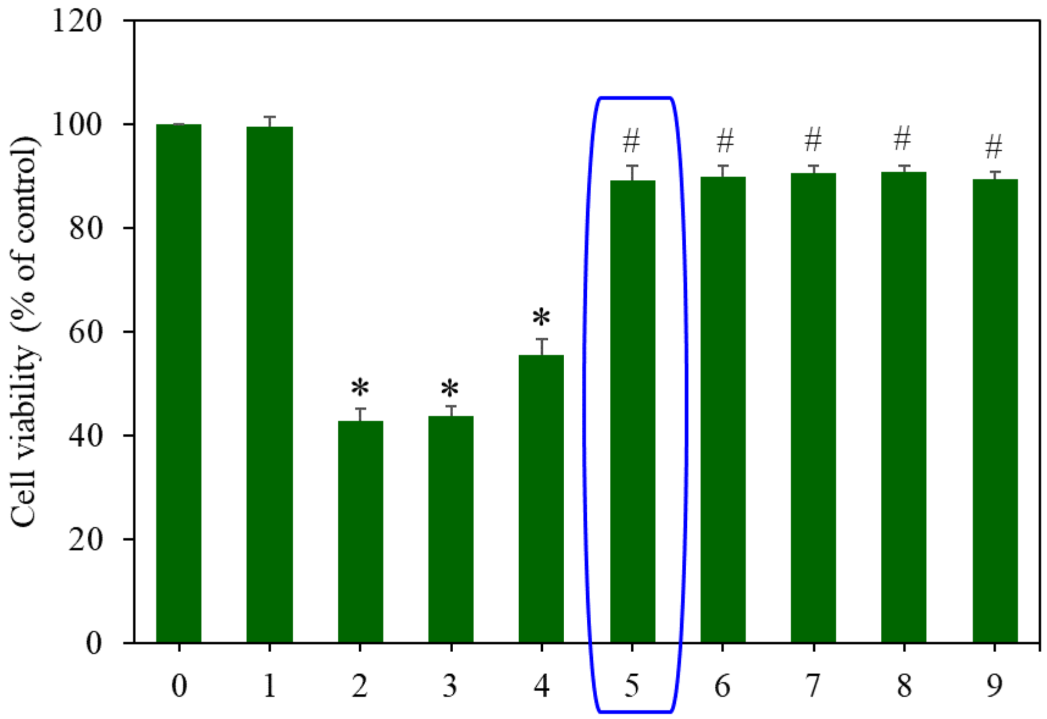

0= Control; 1= 10 µg/ml SWCNTs; 2= 50 µg/ml Pb; 3= 1 µg/ml SWCNTs + 50 µg/ml Pb; 4= 5 µg/ml SWCNTs + 50 µg/ml Pb; **5= 10 µg/ml SWCNTs + 50 µg/ml Pb;** 6= 25 µg/ml SWCNTs + 50 µg/ml Pb; 7= 50 µg/ml SWCNTs + 50 µg/ml Pb; 8= 100 µg/ml SWCNTs + 50 µg/ml Pb; 9= 200 µg/ml SWCNTs+50 µg/ml Pb

Figure S1. Cell viability of A549 cells after co-exposure of SWCNTs and Pb for 24 h. Data provided in this study are represented are mean±SD of three identical experiments made in three replicate. *Significantly different in comparison to the control ($p<0.05$). #Attenuating effects of SWCNTs against Pb-induced cytotoxicity. SWCNTs: Single-walled carbon nanotubes, Pb: Lead, A549 cells: Human lung cells.

References

1. M.A. Majeed Khan, W. Khan, A. Kumar, A.N. Alhazaa, Synthesis of nanosized Cu2O decorated single-walled carbon nanotubes and their superior catalytic activity, Colloids and Surfaces A: Physicochemical and Engineering Aspects. 581 (2019) 123933. https://doi.org/10.1016/j.colsurfa.2019.123933.
2. T. Mosmann, Rapid colorimetric assay for cellular growth and survival: Application to proliferation and cytotoxicity assays, Journal of Immunological Methods. 65 (1983) 55–63. https://doi.org/10.1016/0022-1759(83)90303-4.

3. M. Ahamed, M.J. Akhtar, M.A. Siddiqui, J. Ahmad, J. Musarrat, A.A. Al-Khedhairy, M.S. AlSalhi, S.A. Alrokayan, Oxidative stress mediated apoptosis induced by nickel ferrite nanoparticles in cultured A549 cells, Toxicology. 283 (2011) 101–108. https://doi.org/10.1016/j.tox.2011.02.010.
4. H.A. Alhadlaq, M.J. Akhtar, M. Ahamed, Different cytotoxic and apoptotic responses of MCF-7 and HT1080 cells to MnO2 nanoparticles are based on similar mode of action., Toxicology. 411 (2019) 71–80. https://doi.org/10.1016/j.tox.2018.10.023.
5. M.A. Siddiqui, H.A. Alhadlaq, J. Ahmad, A.A. Al-Khedhairy, J. Musarrat, M. Ahamed, Copper Oxide Nanoparticles Induced Mitochondria Mediated Apoptosis in Human Hepatocarcinoma Cells, PLoS ONE. 8 (2013). https://doi.org/10.1371/journal.pone.0069534.
6. H. Ohkawa, N. Ohishi, K. Yagi, Assay for lipid peroxides in animal tissues by thiobarbituric acid reaction, Analytical Biochemistry. 95 (1979) 351–358. https://doi.org/10.1016/0003-2697(79)90738-3.
7. G.L. Ellman, Tissue sulfhydryl groups, Archives of Biochemistry and Biophysics. 82 (1959) 70–77. https://doi.org/10.1016/0003-9861(59)90090-6.
8. J.T. Rotruck, A.L. Pope, H.E. Ganther, A.B. Swanson, D.G. Hafeman, W.G. Hoekstra, Selenium: Biochemical role as a component of glatathione peroxidase, Science. 179 (1973) 588–590. https://doi.org/10.1126/science.179.4073.588.
9. A.K. Sinha, Colorimetric assay of catalase, Analytical Biochemistry. 47 (1972) 389–394. https://doi.org/10.1016/0003-2697(72)90132-7.
10. M. Ahamed, M.J. Akhtar, H.A. Alhadlaq, Preventive effect of TiO2 nanoparticles on heavy metal Pb-induced toxicity in human lung epithelial (A549) cells, Toxicology in Vitro. 57 (2019) 18–27. https://doi.org/10.1016/j.tiv.2019.02.004.
11. G. Hong, S. Diao, A. L. Antaris, H. Dai, Carbon Nanomaterials for Biological Imaging and Nanomedicinal Therapy. Chem. Rev. 2015, 115, 19, 10816–10906. https://doi.org/10.1021/acs.chemrev.5b00008
